# Robust Identification of Strain Waves due to Low-Velocity Impact with Different Impactor Stiffness

**DOI:** 10.3390/s19061283

**Published:** 2019-03-14

**Authors:** Alessio Beligni, Claudio Sbarufatti, Andrea Gilioli, Francesco Cadini, Marco Giglio

**Affiliations:** Politecnico di Milano, Mechanical Engineering Department, via La Masa 1, 20156 Milano, Italy; claudio.sbarufatti@polimi.it (C.S.); andrea.gilioli@polimi.it (A.G.); francesco.cadini@polimi.it (F.C.); marco.giglio@polimi.it (M.G.)

**Keywords:** low-velocity impacts, strain wave, impactor stiffness, data processing, feature selection, impact identification

## Abstract

Low-velocity impacts represent a major concern for aeronautical structures, sometimes producing barely detectable damage that could severely hamper the aircraft safety, even with regards to metallic structures. For this reason, the development of an automated impact monitoring system is desired. From a passive monitoring perspective, any impact generates a strain wave that can be acquired using sensor networks; signal processing techniques allow for extracting features useful for impact identification, possibly in an automatic way. However, impact wave characteristics are related to the impactor stiffness; this presents a problem for the evaluation of an impact-related feature and for the development of an automatic approach to impact identification. This work discusses the problem of reducing the influence of the impactor stiffness on one of the features typically characterizing the impact event, i.e., the time of arrival (TOA). Two passive sensor networks composed of accelerometers and piezoelectric sensors are installed on two metallic specimens, consisting of an aluminum skin and a sandwich panel, with aluminum skins and NOMEX^TM^ honeycomb core. The effect of different impactor stiffnesses is investigated by resorting to an impact hammer, equipped with different tips. Subsequently, a method for data processing is defined to obtain a feature insensitive to the impactor stiffness, and this method is applied to multiple impact signals for feature uncertainty evaluation.

## 1. Introduction

During the last few decades, structural health monitoring (SHM) systems have become a crucial research topic for the enhancement of the material and structural potential and the reduction of the maintenance and operation costs. In the aeronautical field, a complete SHM system is composed of different sub-systems, with different tasks including the detection of anomalous structural behavior and usage monitoring, comprehensively referred to as Integrated Health and Usage Monitoring Systems (IHUMS). In a damage-tolerant scenario, structures are designed to withstand both service loads, such as those related to a typical operation, and impulsive loads, due to impacts, where typical examples are bird-strikes [1,2], hailstones [1] and debris impacts [3,4].

Focusing on impacts, theoretical knowledge states that impulsive loads generate elastic waves in solid materials [5,6]; those waves are dispersive and the possibility of understanding their behavior has long been of primary interest [7,8]. This aspect is of fundamental importance today, not only due to the difficulty of obtaining simple analytical solutions, but also due to the rapidly increasing use of composite materials in the aeronautical field [9,10,11], even if metallic structures still play a major role. Experimental results, combined with analytical and Finite Element (FE) models, have led to a better understanding of the behavior of dispersive waves [12,13]; those waves are the basis for impact and damage monitoring. Waves can be generated using an actuator or by a foreign object impact event and recorded using a sensor. The former approach is typically referred to as *active monitoring*, targeting the identification of potential damage after an impact event [14], and has been widely applied to various structures, including metallic, honeycomb- [15,16,17] and fiber-reinforced [18] composite components under different sources of damage, such as fatigue, wear, overloads, low- and high-velocity impacts, etc. The latter defines the field of *passive impact monitoring* [11], aiming to identify the occurrence of an impact event, possibly estimating its location [19] and inversely reconstructing the impact force [20] based on signals acquired from passive sensors. In a model-based SHM framework, passive impact monitoring can be used as input to numerical models for damage estimation [14,21], thus triggering the active damage monitoring system for potential real-damage identification.

For passive impact monitoring, the ability to acquire the strain wave is thus of primary importance; different sensor technologies can be adopted: in [7] strain gauges were used to acquire the bending wave generated by an impact of a steel ball on a beam, while in [22,23] the capability and limits of strain gauges for dynamic event acquisition were explained. In [22], the authors used an apparatus to produce strain waves up to 300 kHz in frequency and 2000 *µε* in amplitude and acquired the dynamic signal with strain gauges and laser interferometer, for comparison; gauge length influence, response lag, signal attenuation, static gauge factor variation and other phenomena were analyzed. In [23], the author studied the effect of gauge length on the strain gauge cutoff frequency. In [24], strain-wave-induced accelerations were generated by an impact on a Hopkinson bar and measured by means of accelerometers. The latter was used in [25] to identify external forces and structural damage parameters by means of a sparsity-based reconstruction method. In [26] the strain waves induced by dropping a steel ball on an aluminum plate were recorded using piezoelectric films and acoustic emission sensors; piezoelectric technology for strain acquisition was reviewed in [27]. Finally, in [28] methods for detecting dynamic strain signals with optical fiber (OF) sensors were reviewed, while in [29] an OF-based strain measurement system was tested on-board with its working principle based on a stimulated Brillouin scattering and birefringence phenomena of OF sensors to separate the strain and the temperature effect for wide-area monitoring, i.e., 8 m long full-scale airplane tail-plane. Even microphones were used to identify impact events on CFRP plates [30,31], with a frequency range from 20 Hz to 20 kHz.

However, the ability to acquire the dynamic strain waves is not typically sufficient, in fact, signal processing techniques are necessary to extract the desired information. Several impact identification methods have been developed during the years, mainly based on analytical techniques, model-based techniques and machine learning-based approaches [11,32,33,34,35,36] for impact detection, localization and energy estimation.

To verify the effectiveness of a sensing technology or of an impact identification method, experimental tests are required, and a lot of aerospace materials have been tested: in [37,38] and [39] low-velocity impacts on sandwich structures, fiber-metal laminates and composite materials were reviewed, respectively. During impact tests, not only the structure response, but also the shape of the impacting object [40,41] and the impactor stiffness play key roles in impact and damage identification [8]. This last parameter, together with the energy of the impact, is responsible for the strain wave generation in different frequency ranges, which translate into different wave velocities due to the dispersive nature of wave propagation in panel-like structures, such those considered in this study. Despite this, only a few works in the literature have dealt with the problem of adopting different impactor stiffnesses during the tests: in [42], impact location performance is assessed using a ping pong ball and a rubber ball. In [43], two composite panels were impacted with a hammer equipped with three different tips; in that paper the authors calibrate an inverse method for impact location and force reconstruction using different hammer tips. In [44], an aluminum plate was impacted with six impactors made of different materials, aiming at developing a technique for recognizing the impacting object stiffness. Finally, in [45] an aluminum plate was impacted with a hammer equipped with three different tips, in order to compare the performances of three impact identification techniques, but the training database used for one type of tip did not work for all the tips due to the impact signal frequency content. Some of these examples demonstrate the possibility of performing impact identification in generic scenarios, with different impacting materials leveraging on post-processing algorithms that are mostly insensitive to wave velocity, or training some techniques to cope with related uncertainty. However, at the same time, they show the difficulties encountered when different impactor stiffness and wave velocities are involved, the latter severely hampering the impact identification. Conversely, the focus of the present work is to reduce the direct influence of the different impactor stiffnesses on the impact-related feature of interest, i.e., the time of arrival (TOA) of an elastic wave at the sensor position, before the application of any impact identification technique, the latter being left to future work by the same authors.

To this aim, we propose experimentally investigating the effects of different impactor stiffnesses, reproducing low-velocity impacts and evaluating the velocity of the generated strain waves in correspondence to different impact positions. In order to do so, first, a simple metallic plate and a sandwich panel were chosen as specimens, representative of typical aeronautical structures; this choice then led to the investigation of the influence of different structures on the feature extracted (i.e., the strain wave velocity). Subsequently, different classical sensor technologies were adopted to acquire the dynamic strain signal and evaluate the possibility of extracting features from each sensor network. Finally, the strain wave velocity was assessed by processing the acquired signals; the possibility of extracting a unique feature representative of all the impacts for each specimen tested was also evaluated.

The paper is organized as followed. In Section 2, the experimental setup is described first; then, the preliminary signal analysis and feature selection are presented. Finally, a detailed description of the methods adopted for evaluating the chosen feature and its statistical characterization are presented. In Section 3, the results of the feature evaluation are presented and discussed. Finally, conclusions are drawn in Section 4.

## 2. Materials and Methods

In this section, the experimental setup and the techniques used to obtain the desired results are examined. First, the acquisition system and the equipment are described, then the acquired signals are analyzed and the desired feature is defined. Finally, methods of signal processing and feature extraction are discussed.

### 2.1. Experimental Setup

Two metallic specimens are considered: a simple aluminum plate and a sandwich panel, with aluminum skins and a NOMEX^TM^ (DuPont™, Wilmington, DE, USA) honeycomb core. Their dimensions and mechanical characteristics are listed in Table 1 and Table 2, for the metallic plate and sandwich panel, respectively.

In order to reproduce low-velocity impacts, a PCB mod. 086C03 impact hammer with a steel extender was adopted. In order to reproduce different impactor stiffness, the hammer was equipped with four different tips: a steel tip (PCB 084B03), a Teflon (The Chemours Company, Wilmington, DE, USA) tip (PCB 084B04) and two tips made of two different types of rubber, one softer and one stiffer (PCB 084C11, PCB 084C05). Each specimen was rigidly supported at the four corners and was impacted in 27 different positions with each tip, for a total of 108 impacts, by a single operator.

**Figure 1:**
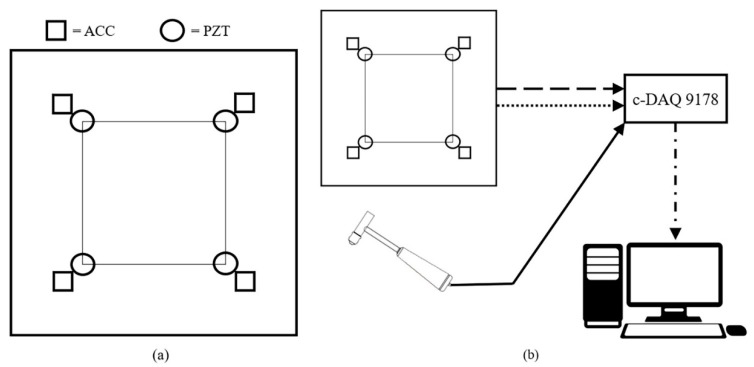
Impact area and sensor positions (**a**) and experimental setup (**b**).

The strain waves generated by the impacts were acquired using two different sensing technologies: accelerometers and piezoelectric sensors. The accelerometers are monoaxial Brüel & Kjær (Nærum, Denmark) DeltaTron type 4508 while the piezoelectric sensors are PIC255 disks with a diameter of 10 mm and thickness 1 mm, produced by PI ceramic (Lederhose, Thuringia, Germany). A total of four sensors for each type are glued at the edges of a 200 × 200 mm square, which is also the limit of the impact area, as shown in Figure 1a.

The hammer signal was acquired by a NI-9234 acquisition card, as well as for all the accelerometer signals. The piezoelectric signals required the adoption of a NI-9229 acquisition card able to acquire signals up to 60 V, due to the capability of the piezoelectric disks to reach high voltage values. All the NI acquisition cards were gathered with a NI c-DAQ-9178 chassis, to simultaneously acquire all the signals using the SignalExpress software (National Instruments, Austin, TX, USA). A trigger, based on the hammer signal, was used and the acquisition frequency was 51.2 kHz. All connections were BNC cables, except for the laptop, which was connected by USB to the c-DAQ chassis. An overview of the entire setup is shown in Figure 1b.

### 2.2. Preliminary Signal Analysis and Feature Extraction

**Figure 2:**
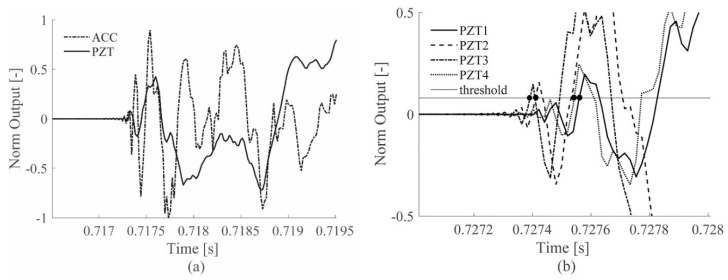
Original signals during an impact event for ACC and PZT sensors, on the aluminum plate (**a**) and the TOA measurement with multiple PZTs on the same aluminum plate (**b**).

Both chosen sensor technologies were able to acquire signals that are representative of the occurring impact phenomenon, however differences in the signal shapes are expected due to the different sensor working principles. In Figure 2a, the comparison of accelerometer (ACC) and piezoelectric (PZT) signal in response of a representative impact shows that both sensor typologies are sensitive to the incoming impact wave, but they also offer different responses.

It can be noted in Figure 2a that both sensors produced a very low baseline signal, including the environmental and sensors uncertainties before the occurrence of an impact, thus facilitating the identification of the impact event.

With this aim, the feature chosen to represent the impact event is the TOA of the elastic wave to the sensors, defined as the time at which the sensor signal exceeds a properly defined threshold value for the first time. Figure 2b shows the TOAs identified by the PZT sensors (black dots) for a representative impact on the aluminum plate, using non-filtered signals.

However, although a stable baseline is visible in Figure 2a, the TOA could not be identified regardless of the properties of the impacted structure, the impactor, the impact energy and the sensor dynamic behavior, which influence the frequency content of the signal. In fact, the strain waves generated by impacts are known from the theory to be multiple flexural dispersive waves [5]. Different strain wave components are generated after the impact, associated to different frequencies, different amplitudes and travelling at different speeds. Thus, the same wave front should be identified by each sensor for a correct selection of the TOA and an efficient passive impact identification. In fact, the distance of the impact position from a sensor determines the TOA of the wave with respect to the sensor itself; the sensor closest to the impact location has the lowest TOA for the same wave front, while the most distant sensor has the largest TOA. The sensors can be listed in ascending TOA order, creating a sensor sequence. The performances of the passive impact identification methodology are strongly related to the capability of defining the correct TOA and thus the correct sensor sequence. Errors in TOA evaluation could severely hamper the methodology’s accuracy. For these reasons, it is necessary to process the original data to obtain a more regular signal, before impact identification.

### 2.3. Frequency Analysis

**Figure 3:**
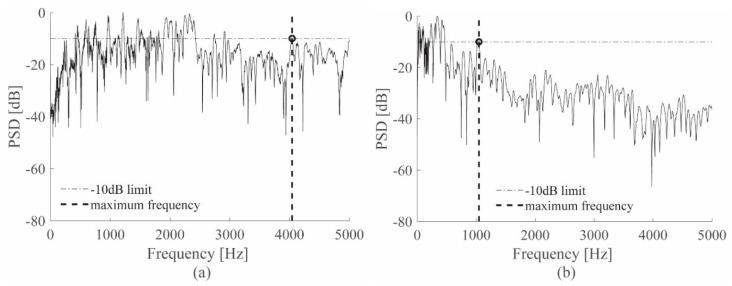
−10 dB extraction procedure for ACC (**a**) and PZT (**b**) sensors.

Considering the different physical quantities measured by the PZT and ACC, a frequency analysis was performed to compare the sensors and to identify the frequency range conveying significant information. First, the one-sided power spectral density (PSD) was computed for all the sensors, for each impact position and each specimen and its maximum peak was set to zero; then, the maximum impact frequency was evaluated, imposing a limit equal to −10 dB and selecting the highest frequency that crosses this limit [5]. The −10 dB value was chosen to obtain a frequency for which 90% of the signal energy is considered, thus most of the impact energy was contained in the frequencies from zero to the maximum impact frequency estimated. One example is reported in Figure 3a,b, where the −10 dB limit was extracted from the ACC and PZT sensor signal during a representative impact, respectively.

After evaluating the significant impact frequency for each sensor on each specimen and for each impact, a normality test, i.e., the Lilliefors Test [46], was executed in Matlab using the function *lillietest*. Table 3 summarizes the Lilliefors normality test results; the result *H* = 1 indicates that the null hypothesis of normal distribution was rejected at the 5% level of significance.

**Figure 4:**
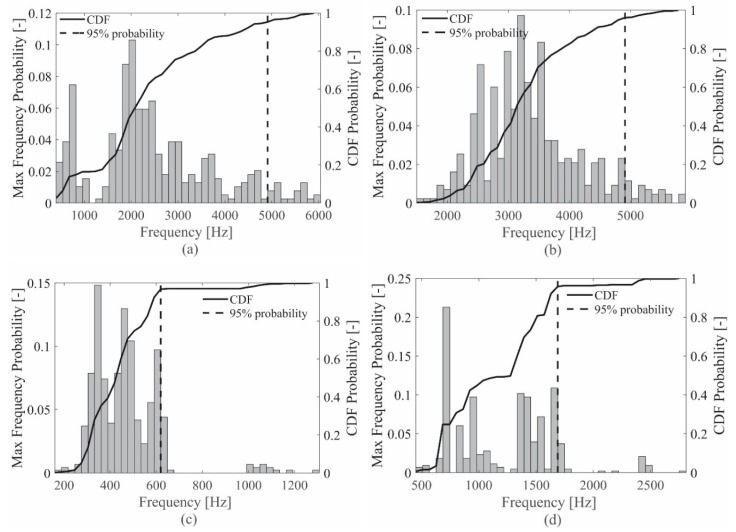
CDF and 95% limit for the ACC on the aluminum plate (**a**) and the sandwich panel (**b**) and for the PZT on the aluminum plate (**c**) and the sandwich panel (**d**).

The results show that the distribution of the maximum impact frequencies for each sensor on each specimen cannot be assumed to be Gaussian with sufficient confidence. Thus, the cumulative distribution function (CDF) was used to define the cutoff sensor frequency for the acquired signal on each specimen, specifically considering the 95% of the CDF as the limit frequency. Figure 4a,b shows the procedure results for the ACC sensor, while Figure 4c,d shows the results for the PZT sensor, on the aluminum plate and the sandwich panel, respectively.

For the ACC case, a unique frequency can be considered since the results were very similar for both the specimens (Figure 4a,b), i.e., 4912 Hz. On the other hand, two different characteristic frequencies were evaluated for the PZT case on the two specimens (Figure 4c,d), i.e., 619 Hz for the skin plate and 1689 Hz for the sandwich panel.

At the end of the frequency analysis, the two main results are: (i) the limit sensor signal frequency reached during the tests for the ACC sensor is 4912 Hz, valid for both the specimens, while (ii) a limit sensor frequency of 619 Hz and 1689 Hz is reached by the PZT sensor on the aluminum plate and the sandwich panel, respectively.

In addition to these values, 8000 Hz and 2000 Hz were also considered significant threshold frequencies. At the maximum frequency of 8000 Hz the ACC sensor still showed a linear response, as reported in the producer certification. For both the ACC and PZT sensors the 2000 Hz value was chosen as the theoretical maximum frequency reachable using an impact hammer identical to the one used during the test [47]. All significant cutoff frequencies for both sensors on each specimen are summarized in Table 4.

Finally, assuming all the frequencies previously discussed, a low pass Infinite Impulse Response (IIR) filter was applied to the signals using Matlab; considering that IIR filters induce some time delay, the Matlab function *filtfilt* was used to compensate for this effect and avoid any filter influence on the TOA assessment.

### 2.4. Threshold Selection

Considering the problems of selecting the baseline threshold discussed in Section 2.2, two different approaches were chosen for the extraction of the TOA. The first one was a visual approach, in which the TOA was identified visually for each impact, manually selecting the baseline threshold. This was done in order to obtain a reference result that is as unaffected as possible by the unavoidable errors related to the dispersive nature of the strain waves. In fact, if a visual identification of the TOA was performed, the signal was observed and the threshold exceedance point was selected in a consistent manner, referring to the occurrence of the same wave front at different sensors, thus guaranteeing the correctness of the sequence at which the strain waves reach the sensors. This second point is of great importance in the impact identification procedure, as is highlighted in the following. However, this visual approach is highly time consuming, the results could be affected by human errors and the methodology does not permit the definition of a fixed threshold value for an automatic impact detection algorithm, which is a desired result in any structural health monitoring application.

For this reason, a second approach is proposed, namely an automatic TOA evaluation, for which the threshold was selected based on the percentage of correct sensor sequences, considering all the impact positions on each specimen. First, the signals were gathered considering the sensor technology and the specimens, then they were normalized, dividing the sensor’s output by the maximum peak reached during each impact. Finally, the threshold was varied from the minimum value (−1) to the maximum value (+1) and, in correspondence to each threshold value, the sequence accuracy was evaluated as:

(1) $$sequence\;accuracy=\frac{number\;of\;correct\;sequences}{number\;of\;impacts}.$$

**Figure 5:**
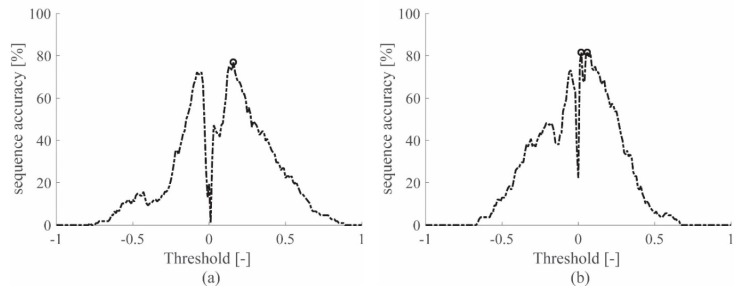
Sequence accuracy of the ACC signals on the aluminum plate (**a**) and the sandwich panel (**b**), when the 0–619 Hz or 0–1689 Hz filter is applied, respectively.

**Figure 6:**
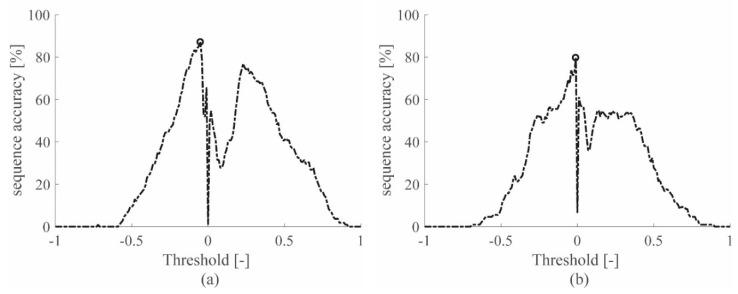
Sequence accuracy for the PZT signals on the aluminum plate (**a**) and the sandwich panel (**b**), when the 0–619 Hz or 0–1689 Hz filter is applied, respectively.

The sequence accuracy resulting for each threshold value is drawn in Figure 5 for the accelerometers and in Figure 6 for the piezoelectric sensors. The main outcome of this procedure is the threshold value selection to be used in the automatic approach, for each sensor technology on each specimen. Using the selected threshold value, guarantees the higher sequence accuracy for the impact database considered, for the automatic TOA evaluation.

Note that Figure 5 and Figure 6 show only the results obtained for the signals filtered with the lowest cutoff frequency among those available in Table 4, specifically with low-pass ranges 0–619 Hz and 0–1689 Hz for the aluminum plate and sandwich panel, respectively. By considering only the lowest cutoff frequencies, the best sequence accuracy results were obtained. The interested reader can refer to Table A1 in the Appendix A, where the performances obtained with all the low-pass filters adopted are reported for both specimens.

### 2.5. TOA Data Processing

In order to simulate a real impact monitoring scenario, where no trigger of the impact instant is available, a relative *TOA* is presented hereafter, defined as the difference (Δ*T*) between the *TOA* of the *i*-th sensor (*TOA_i_*) and the *TOA* of the first sensor reached by the impact wave (*TOA*_0_).

(2) $$\Delta T_{i}=TOA_{i}-TOA_{0}\;\;i=1,2,\ldots ,N-1,$$

where *N* is the number of sensors for impact identification.

Similar considerations apply for the impact distance. Assuming the impact location as a known variable, thus neglecting the error in impact location due to the operator, a relative distance (Δ*L*) was computed as the difference between the *i*-th sensor distance from the impact location (*D_i_*) and the distance from the same impact location of the first sensor reached by the impact wave (*D*_0_).

(3) $$\Delta L_{i}=D_{i}-D_{0}\;\;i=1,\;2,\ldots ,\;N-1$$

**Figure 7:**
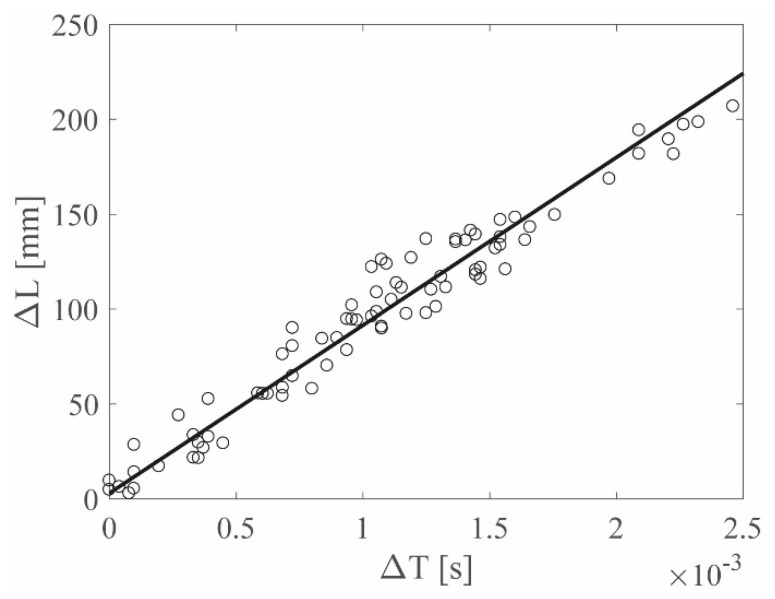
(Δ*L*–Δ*T*) graph example.

The *N−1* pairs of values (Δ*L*–Δ*T*) are available after each impact and, after repeating the impact *K* times for *K* different locations, *K*(*N−1*) (Δ*L*–Δ*T*) couples were collected in a graph as shown in Figure 7. The linearity between the relative distance and the relative *TOA* is evident and a linear regression was performed. The choice of representing the results within a unique, coherent framework was made to show that different impactor stiffnesses excite different wave propagation modes, which, in turn, are associated to different frequencies, thus inducing different wave velocities. In fact, the slope of the linear regression in the (Δ*L*–Δ*T*) graph was used to represent the wave velocity.

Regarding the linear regression, first the correlation between the Δ*L* and Δ*T* data was checked and the Pearson coefficient was evaluated for all the datasets; Table A2 in the Appendix A reports the results obtained using the manual approach, for the ACC and PZT sensors, respectively. All of the Pearson coefficients were close to 1 and all the associated *p*-values were approximately zero, suggesting that a linear correlation was present for the (Δ*L*–Δ*T*) data, thus justifying the use of the linear regression for the data representation. The same conclusions apply for the data collected by the automatic approach with the results listed in Table A2 in the Appendix A.

An outlier analysis was then used to eliminate data that were not representative of the studied phenomenon, due to errors in the experimental tests’ execution. The presence of outliers is related to the modality chosen for the test execution and for the *TOA* evaluation. In fact, all the impacts were executed manually, resulting in a potentially different real impact position from the target impact location, inducing errors on the Δ*L* parameter. The same considerations were made for the Δ*T* parameter; even when it was evaluated based on the visual approach, signal misinterpretation could happen due to the difficulty of distinguishing the various strain wave contributions associated to different frequencies and velocities within the time series. Also, sensors or acquisition system failures could give results not representative of the impact phenomenon. These facts induce errors in the Δ*L*–Δ*T* data that may give rise to outliers.

As a consequence, the (Δ*L*–Δ*T*) data displayed a certain amount of dispersion and, even though the linear regression visibly appeared to be a good model for the (Δ*L*–Δ*T*) dataset representation, it required verification that the three hypotheses for the simple linear regression are valid, namely (i) the normality of the residuals, (ii) the homoscedasticity and (iii) the independence of the residuals. For brevity’s sake, the hypotheses were verified for the majority of the analyzed datasets, thus assuming that the simple linear regression is adequate for modeling the (Δ*L*–Δ*T*) data:

(4) ΔL=mΔT+q,

where Δ*L* and Δ*T* were evaluated from the experimental dataset, *m* is the slope and *q* the intercept of the linear regression line. Finally, the uncertainties related to the linear regression parameters, i.e., slope *m* and intercept *q*, could be evaluated as follows:

(5) $$\sigma_{m}=\sqrt{\frac{n}{n\sum \Delta T_{i}^{2}-{\left(\sum \Delta T_{i}\right)}^{2}}}$$

(6) $$\sigma_{q}=\sqrt{\frac{\sum \Delta T_{i}^{2}}{n\sum \Delta T_{i}^{2}-{\left(\sum \Delta T_{i}\right)}^{2}}},$$

where $\sigma_{m}$ and $\sigma_{q}$ represent the slope and intercept standard deviations, respectively, and *n* is the total number of samples included in the analysis.

## 3. Results and Discussion

In this section, the results are presented for the metallic plate first, then for the sandwich panel; a comparison between the ACC and PZT results is shown for all the low-pass filters adopted. Then the automatic approach results are presented. The linear regression parameter uncertainties were evaluated for all cases.

### 3.1. Aluminum Plate Results

For the first part of the work, the Aluminum plate signals were analyzed using the visual approach. In this case the feature TOA and thus the parameter Δ*T*, were evaluated by visually determining the instant at which the impact wave arrived at the sensor.

**Figure 8:**
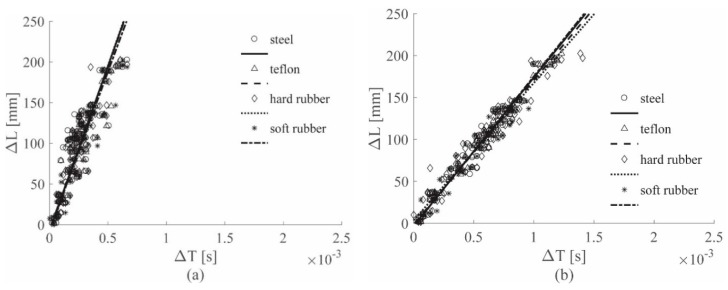

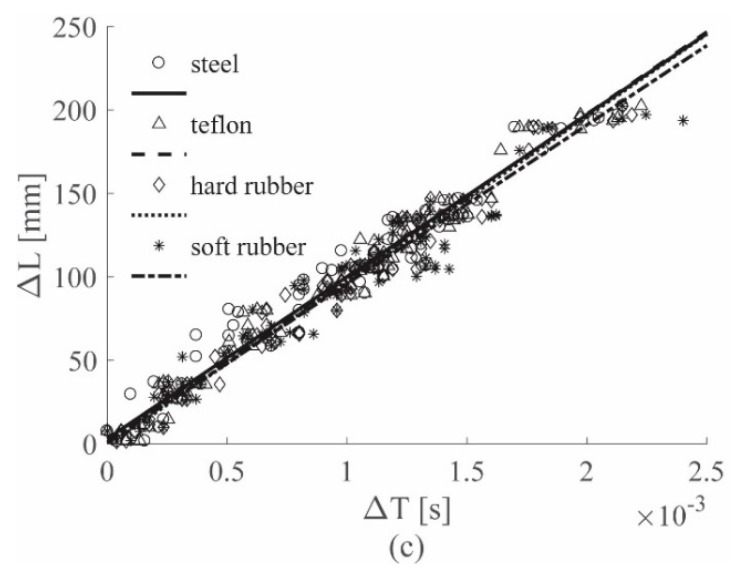
(Δ*L*–Δ*T*) visual approach results for the ACC sensors, Al plate, all tips, filtered with the 0–4912 Hz filter (**a**), 0–2000 Hz filter (**b**) and 0–619 Hz filter (**c**).

**Figure 9:**
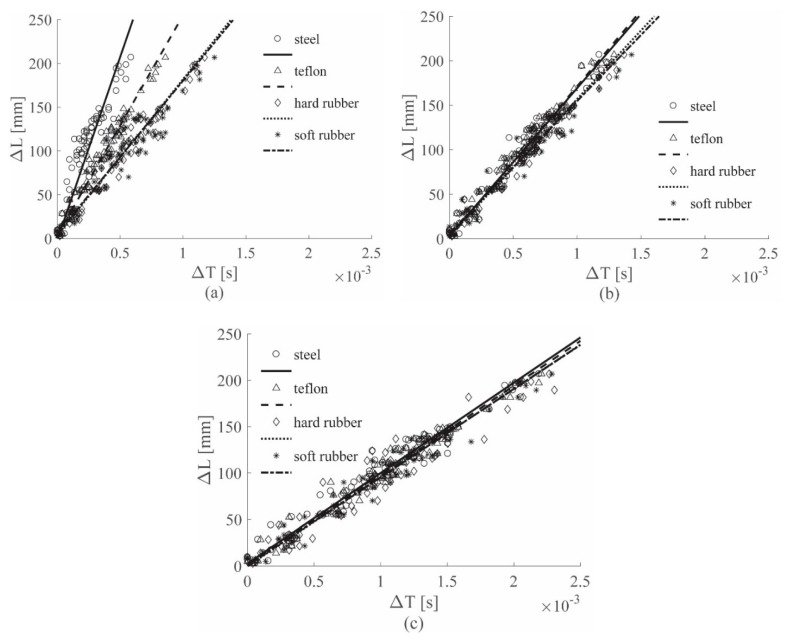
(Δ*L*-Δ*T*) visual approach results for the PZT sensors, Al plate, all tips, filtered with the 0–4912 Hz filter (**a**), 0–2000 Hz filter (**b**) and 0–619 Hz filter (**c**).

**Figure 10:**
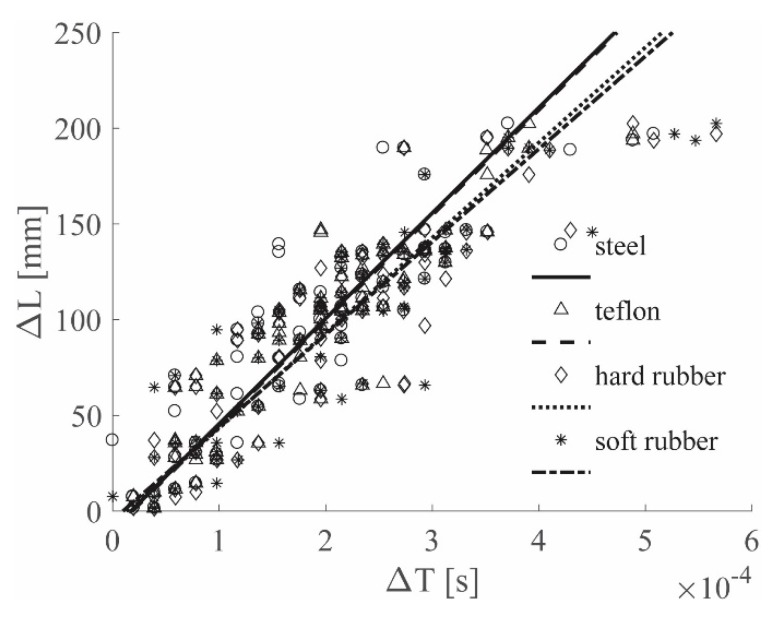
(Δ*L*–Δ*T*) visual approach results for ACC sensors, Al plate, all tips, filtered with the 0–8000 Hz filter.

Figure 8 shows the results for the ACC sensors, while Figure 9 shows the results for the PZT sensors; both figures show the 0–4912 Hz low-pass filter results for all the hammer tips, in position (a). Position (b) refers to the 0–2000 Hz low-pass filter results, while position (c) to the 0–619 Hz low-pass filter results. Finally, Figure 10 depicts the results for the 0–8000 Hz range, for the ACC sensor only. For simplicity of interpretation, the linear regressions are also reported and used as references; different colors are used to identify the different tips used.

Figure 9a and Figure 10 show the results for ACC and PZT, respectively, for which the widest frequency range was adopted for each sensor, i.e., 0–8000 Hz for ACC and 0–4912 Hz for PZT. In both images different lines have different slopes; considering that each colored line represents a different hammer tip, it is possible to state that the impactor stiffness significantly influences the (Δ*T*–Δ*L*) relationship.

In Figure 8a, the cutoff frequency estimated for the ACC with the approach introduced in Section 2.3 was used; in this case, a slope similarity was visible for all the lines. The impactor stiffness failed to influence the results if the estimated cutoff frequency for the ACC sensors was adopted.

Figure 8b and Figure 9b show the results for the 0–2000 Hz range for the ACC and PZT sensors, respectively; lower cutoff frequencies resulted in slower waves—in fact, the line slopes were lower in both the cases if compared to those ones obtained with higher cutoff frequencies. Moreover, the difference between the lines in the PZT case decreased, indicating that a reduction of the filtering frequency causes a reduction in the impactor stiffness influence on the wave velocity.

Finally, Figure 8c and Figure 9c show the results for the 0–619 Hz range for the ACC and PZT sensors, respectively. The adopted frequency is the cutoff frequency estimated with the approach in Section 2.3 for analyzing PZT signals; thus from Figure 9c, a significant impactor stiffness influence reduction was obtained for the PZT results. The same conclusion was reached from Figure 9a, in which the ACC results were obtained by adopting the cutoff frequency estimated analyzing the ACC signals. Moreover, the results obtained with the ACC and the PZT sensors showed very similar slopes.

### 3.2. Sandwich Panel Results

**Figure 11:**
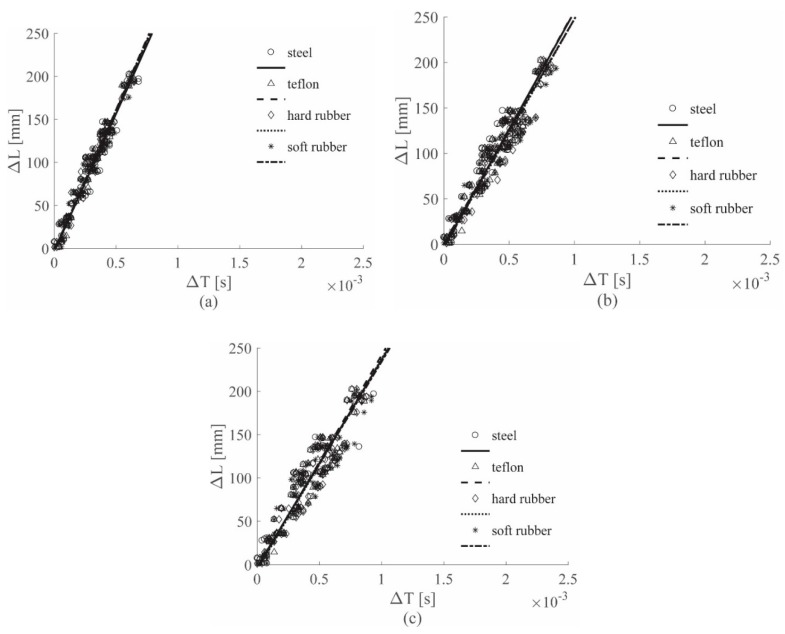
(Δ*L*–Δ*T*) visual approach results for the ACC sensors, sandwich panel, all tips, filtered with the 0–4912 Hz filter (**a**), 0–2000 Hz filter (**b**) and 0–1689 Hz filter (**c**).

**Figure 12:**
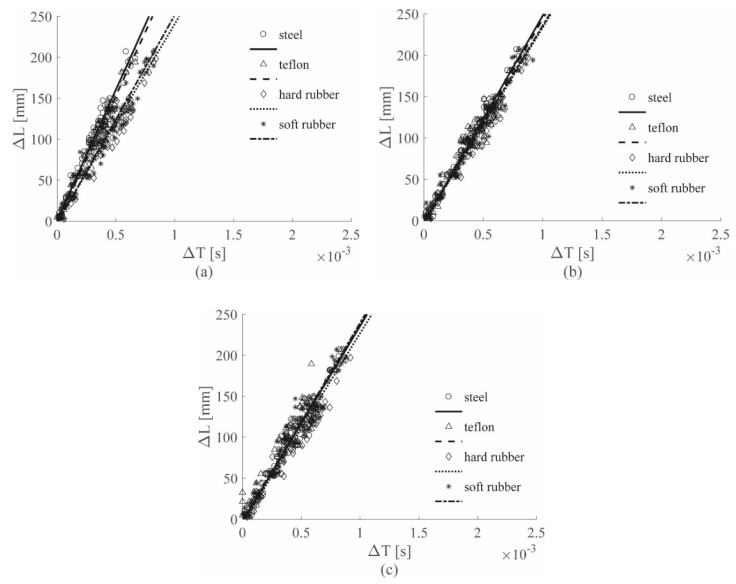
(Δ*L*–Δ*T*) visual approach results for the PZT sensors, sandwich panel, all tips, filtered with the 0–4912 Hz filter (**a**), 0–2000 Hz filter (**b**) and 0–1689 Hz filter (**c**).

**Figure 13:**
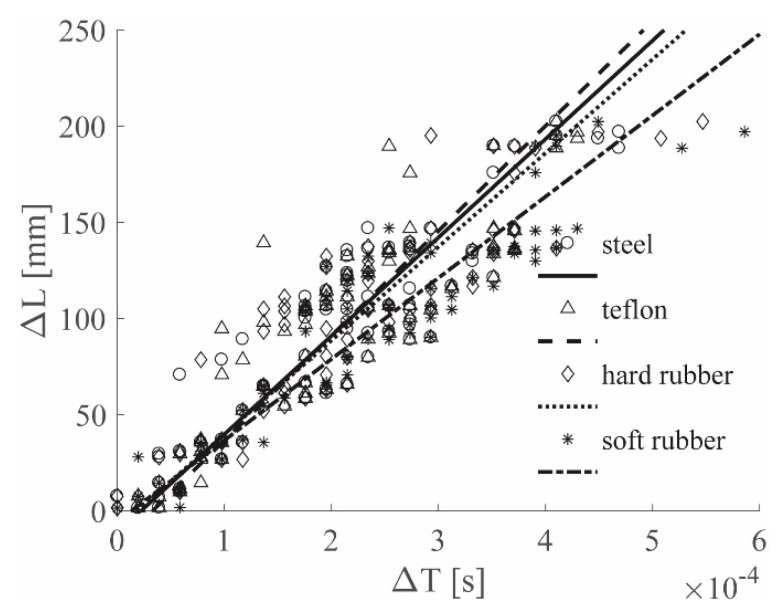
(Δ*L*–Δ*T*) visual approach results for the ACC sensors, sandwich panel, all tips, filtered with the 0–8000 Hz filter.

Figure 11 shows the visual approach results for the ACC sensor, while Figure 12 gives those for the PZT sensor; in these figures, position (a) refers to the 0–4912 Hz low-pass filter, position (b) to the 0–2000 Hz low-pass filter, and position (c) to the 0–1689 Hz low-pass filter results. Figure 13 depicts the results for the 0–8000 Hz range, for the ACC sensor only; linear regression lines were used as the references for all the conclusions.

Figure 13 also shows that, considering the sandwich panel case, in the 0–8000 Hz filtering range, different hammer tips correspond to lines with different slopes; the same is visible in Figure 12a, which refers to the results obtained filtering the PZT signals with the wider range, i.e., 0–4912 Hz. Thus, also for the sandwich panel, different hammer tips generated strain waves with different velocities, as well as for the aluminum plate case.

In Figure 11a, the cutoff frequency defined for the ACC was used, resulting in similar slopes for all the lines; thus, the impactor stiffness has no influence on the ACC results if the estimated cutoff frequency for the ACC case was adopted.

Figure 11b and Figure 12b illustrate the results for the 0–2000 Hz range for the ACC and the PZT sensors, respectively; in the sandwich panel case, as well as for the aluminum plate case, the cutoff frequency reduction caused a decrease in the wave velocity, i.e., the slope coefficient *m*, as shown in Table 5 below for the ACC case.

Finally, Figure 11c and Figure 12c show the results for the 0–1689 Hz range for the ACC and the PZT sensors, respectively. For the sandwich panel case, as well as for the aluminum plate case, the lowest cutoff frequency was the value estimated with the approach in Section 2.3, for the PZT sensors. For the PZT results in Figure 12c, a strong impactor stiffness influence reduction was observed, similar to that obtained in Figure 11a with ACC, adopting the cutoff frequency value estimated by analyzing the ACC signals. Moreover, results obtained with the ACC and the PZT sensors, for the 0–1689 Hz range, showed similar slopes, as also seen in the aluminum plate case.

Note that for the sandwich panel the 2000 Hz limit was very close to the lowest cutoff frequency, i.e., 1689 Hz; for this reason, the impactor stiffness influence reduction was already observed in the PZT case in Figure 12b for the 0–2000 Hz range.

For this first part of the work, a low-pass filter that adopts the cutoff frequency evaluated with the approach introduced in Section 2.3 was applied to reduce the influence of the impactor stiffness.

Another fundamental factor that significantly affects the results is related to the stiffness of the impacted structure; in fact, structures with different stiffness produce strain waves with different velocities. Considering the results obtained with the cutoff frequency 0–619 Hz for the aluminum plate and 0–1689 Hz for the sandwich panel for the ACC case (see Figure 8c and Figure 11c), the line groups showed different slopes. Similar conclusions can be drawn by observing the results for the PZT case (see Figure 9c and Figure 12c). Thus, it can be concluded that by processing the signal with a filter built according to the approach introduced in Section 2.3, the influence of the impactor stiffness was reduced, while the influence of the impacted structure stiffness remained unaltered.

Finally, for the results obtained in Section 3.1 and Section 3.2 it is possible to conclude that:

- Different impactor stiffnesses produce different impact wave velocities, represented as line slopes in the plots.
- Frequencies identified with the approach introduced in Section 2.3 are suitable for the reduction of the impactor stiffness influence on the impact wave velocity evaluation, for both sensors considered.
- Frequencies identified with the approach introduced in Section 2.3 are unsuitable for the reduction of the impacted structure stiffness influence on the impact wave velocity evaluation, for both sensors considered.

Due to the impactor stiffness influence reduction, unique datasets were created gathering all the tip results, for the aluminum plate 0–619 Hz filtered, and for the sandwich panel, 0–1689 Hz filtered. Thus, a unique linear regression representative of all the impactor stiffness cases could be calculated and the linear regression parameter uncertainties could be evaluated. The results are listed in Table A3 in the Appendix A.

### 3.3. Automatic Approach Results

The automatic approach was considered in the second part of the work. In this case the feature TOA, and consequently also the parameter Δ*T*, were evaluated choosing a threshold value according to the procedure explained in Section 2.4 and then by selecting the correct points using an automated algorithm. Only the cases 0–619 Hz and 0–1689 Hz were considered, not only because of the impactor stiffness influence reduction, as just shown, but also for the higher level of sequence accuracy that could be obtained using those frequencies, as shown in Section 2.4.

**Figure 14:**
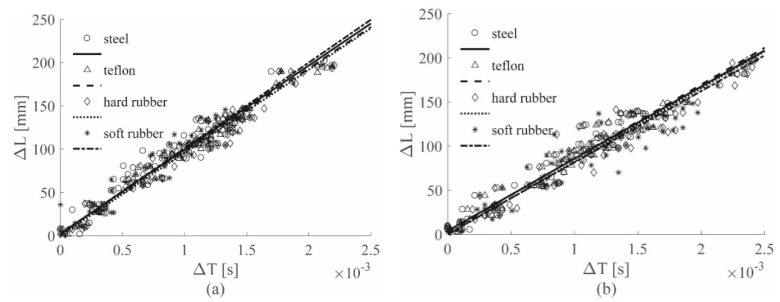
(Δ*L*–Δ*T*) automatic approach results for the Al plate, all tips, the ACC sensors (**a**) and the PZT sensors (**b**), both filtered with the 0–619 Hz filter.

Figure 14 shows the results obtained on the aluminum plate, for the ACC and PZT sensors respectively. The results are consistent with the results obtained visually shown in Figure 8c and Figure 9c.

**Figure 15:**
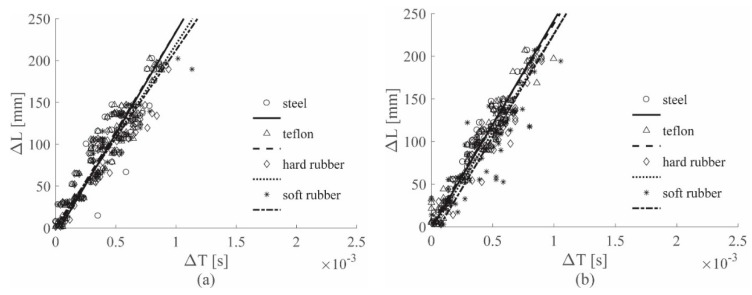
(Δ*L*–Δ*T*) automatic approach results for sandwich panel, all tips, ACC sensors (**a**) and PZT sensors (**b**), both filtered with the 0–619 Hz filter.

Figure 15 shows the results obtained on the sandwich panel, for the ACC and PZT sensors, respectively. The results are very similar to those obtained manually in Figure 11c and Figure 12c. For both the visual and the automated approach, the specimen stiffness influence could not be removed from the results.

Thus, conclusions identical to those gleaned from the visual approach of Section 3.1 and Section 3.2 were drawn.

As before, in this case a unique dataset was created to calculate a unique linear regression and then to evaluate the linear regression parameter uncertainties. The results are listed in Table A4 in the Appendix A. The comparison of the results in Table A3 and Table A4 in the Appendix A shows that the automatic approach allowed for obtaining standard deviation values similar to those obtained with the visual approach. In fact, for the aluminum plate the automatic approach showed an increment in the slope standard deviation equal to 9% and 4%, for the ACC sensors and the PZT sensors, respectively, whereas the sandwich panel showed an increment equal to 21% and 33%, for the ACC sensors and the PZT sensors, respectively.

## 4. Conclusions

In this work, a methodology for the reduction of the impactor stiffness influence on the evaluation of the impact strain wave time of arrival (TOA) and velocities has been proposed. The procedure can be summarized as follows:

- First, a test setup was developed to acquire impact strain waves using two typical sensor technologies for the aeronautical field, i.e., accelerometers and piezoelectric sensors. Different impactor stiffnesses were reproduced using an impact hammer equipped with different tips.
- The procedure cutoff frequency was evaluated analyzing the acquired signals and was subsequently applied by post-processing the signals.
- The TOA feature was evaluated to estimate the strain wave velocities, using the post-processed signals. Two approaches were used, a visual and an automatic approach; in the second case the threshold had to be carefully chosen to develop the automatic algorithm for feature extraction.

Finally, the results were interpreted using a linear regression model and the linear regression parameter uncertainties were evaluated. The first part of the work confirmed that different impactor stiffnesses induce different impact wave velocities and that a cutoff frequency could therefore be defined and used for signal processing to obtain results that are not affected by the impactor stiffness. On the contrary, the impact wave velocity dependence was not reduced due to different specimen stiffnesses.

In the second part of the work an automatic approach was developed; it showed good capability to determine the correct sensor sequence and good accordance with the visual approach for evaluating the impact wave velocity. This approach could be useful for defining a procedure for the development of an automated impact monitoring system able to neglect the impactor stiffness in identifying the impact event. However, further work is required on the automatic TOA evaluation procedure to enhance its performance, especially considering the sequence accuracy.

Finally, all the results were represented using a linear regression and its parameter uncertainties, i.e., slope *m* and intercept *q* standard deviations. Those uncertainties are essential to evaluate the error propagation related to impact wave velocity evaluation errors, if we consider the impact monitoring system as part of a wider structural health monitoring system.

## Figures and Tables

**Table 1:** Dimensions and mechanical characteristics of the metallic plate.

Material	Al2024-T3
Dimensions	400 × 400 × 1.5 mm
Density	2780 Kg/m^3^
Young Modulus	73.1 GPa
Poisson coeff.	0.33

**Table 2:** Dimensions and mechanical characteristics of the sandwich panel.

Dimensions	400 × 400 × 22.05 mm
Skin material	Al2024-T3
Skin thickness	1.5 mm (×2 skins)
Core material	HRH10-3/16-2
Core thickness	19.05 mm
Core density	32 Kg/m^3^

**Table 3:** Lilliefors normality test results.

	ACC—Al Plate	PZT—Al Plate	ACC—Sandwich	PZT—Sandwich
**H**	1	1	1	1

**Table 4:** Identified significant cutoff frequencies for the ACC and PZT sensor, on both specimens.

	ACC—Al Plate	PZT—Al Plate	ACC—Sandwich	PZT—Sandwich
Frequency [Hz]	8000	-	8000	-
	4912	4912	4912	4912
	2000	2000	2000	2000
	619	619	1689	1689

**Table 5:** Linear regression slope coefficient *m* for the ACC sensor, 0–4912 Hz and 0–2000 Hz cases.

Frequency [Hz]	Linear Regression Slope Coefficient *m—*ACC Sensors—[m/s]			
	Steel	Teflon	Hard Rubber	Soft Rubber
0–4912	320.1	331.1	326.5	323.9
0–2000	258.4	261.6	251.1	247.0

## Appendix A

**Table A1:** Sequence accuracy overview.

	ACC [%]	PZT [%]	Frequency [Hz]
**Aluminum Plate**	59.26	-	8000
	57.41	75.00	4912
	62.04	76.85	2000
	76.85	87.04	619
**Sandwich Panel**	55.56	-	8000
	67.59	79.63	4912
	81.48	82.41	2000
	82.41	83.33	1689

**Table A2:** Pearson coefficients for the datasets obtained with the ACC and PZT sensor, for the visual and the automatic approach.

4912 Hz—Visual	ACC	PZT	Hammer Tip
**Aluminum Plate**	0.8856	0.9011	Steel
	0.8989	0.9863	Teflon
	0.8956	0.9699	Hard rubber
	0.8910	0.9527	Soft rubber
**Sandwich Panel**	0.9715	0.9884	Steel
	0.9857	0.9894	Teflon
	0.9783	0.9706	Hard rubber
	0.9807	0.9659	Soft rubber
**2000 Hz—Visual**	**ACC**	**PZT**	**Hammer Tip**
**Aluminum Plate**	0.9804	0.9779	Steel
	0.9921	0.9874	Teflon
	0.9635	0.9818	Hard rubber
	0.9650	0.9768	Soft rubber
**Sandwich Panel**	0.9574	0.9876	Steel
	0.9647	0.9701	Teflon
	0.9601	0.9770	Hard rubber
	0.9379	0.9802	Soft rubber
**619 Hz/1689 Hz—Visual**	**ACC**	**PZT**	**Hammer Tip**
**Aluminum Plate**	0.9766	0.9736	Steel
	0.9828	0.9765	Teflon
	0.9837	0.9709	Hard rubber
	0.9684	0.9638	Soft rubber
**Sandwich Panel**	0.9339	0.9782	Steel
	0.9445	0.9463	Teflon
	0.9425	0.9642	Hard rubber
	0.9366	0.9667	Soft rubber
**619 Hz/1689 Hz—Automatic**	**ACC**	**PZT**	**Hammer Tip**
**Aluminum Plate**	0.9630	0.9800	Steel
	0.9850	0.9798	Teflon
	0.9804	0.9588	Hard rubber
	0.9701	0.9479	Soft rubber
**Sandwich Panel**	0.9148	0.9726	Steel
	0.9394	0.9585	Teflon
	0.9047	0.9549	Hard rubber
	0.9457	0.8860	Soft rubber

**Table A3:** Linear regression coefficient uncertainties; visual approach.

	ACC		PZT		Frequency [Hz]
	σ_m_ [V]	σ_q_ [V]	σ_m_ [V]	σ_q_ [V]	
**Aluminum Plate**	1.260 × 10^7^	1.386 × 10^5^	1.812 × 10^7^	1.989 × 10^5^	0–619
**Sandwich Panel**	8.511 × 10^8^	9.367 × 10^6^	6.917 × 10^8^	7.547 × 10^6^	0–1689

**Table A4:** Linear regression coefficient uncertainties; automatic approach.

	ACC		PZT		Frequency [Hz]
	σ_m_ [V]	σ_q_ [V]	σ_m_ [V]	σ_q_ [V]	
**Aluminum Plate**	1.378 × 10^7^	1.465 × 10^5^	1.892 × 10^7^	1.976 × 10^5^	0–619
**Sandwich Panel**	1.066 × 10^7^	1.130 × 10^5^	9.246 × 10^8^	1.010 × 10^5^	0–1689
